# A Visualization Method of Knowledge Graphs for the Computation and Comprehension of Ultrasound Reports

**DOI:** 10.3390/biomimetics8080560

**Published:** 2023-11-21

**Authors:** Jiayi Feng, Runtong Zhang, Donghua Chen, Lei Shi

**Affiliations:** 1 Department of Information Management, Beijing Jiaotong University, Beijing 100044, China; 18113057@bjtu.edu.cn; 2 Department of Information Management, University of International Business and Economics, Beijing 100029, China; dhchen@uibe.edu.cn; 3 School of Computing, Newcastle University, Newcastle upon Tyne NE4 5TG, UK; lei.shi@ncl.ac.uk

**Keywords:** ultrasound report, knowledge graph, knowledge representation, machine learning, natural language processing, precision medicine

## Abstract

Knowledge graph visualization in ultrasound reports is essential for enhancing medical decision making and the efficiency and accuracy of computer-aided analysis tools. This study aims to propose an intelligent method for analyzing ultrasound reports through knowledge graph visualization. Firstly, we provide a novel method for extracting key term networks from the narrative text in ultrasound reports with high accuracy, enabling the identification and annotation of clinical concepts within the report. Secondly, a knowledge representation framework based on ultrasound reports is proposed, which enables the structured and intuitive visualization of ultrasound report knowledge. Finally, we propose a knowledge graph completion model to address the lack of entities in physicians’ writing habits and improve the accuracy of visualizing ultrasound knowledge. In comparison to traditional methods, our proposed approach outperforms the extraction of knowledge from complex ultrasound reports, achieving a significantly higher extraction index (η) of 2.69, surpassing the general pattern-matching method (2.12). In comparison to other state-of-the-art methods, our approach achieves the highest P (0.85), R (0.89), and F1 (0.87) across three testing datasets. The proposed method can effectively utilize the knowledge embedded in ultrasound reports to obtain relevant clinical information and improve the accuracy of using ultrasound knowledge.

## 1. Introduction

Medical imaging techniques, such as ultrasound examination, X-ray, computed tomography, and magnetic resonance imaging, offer great value in clinical diagnosis [1,2]. Medical experts typically document various types of examination data using free text in their natural language into a report to describe patients’ symptoms, conditions, imaging findings, and even diagnosis results [3]. These narrative reports based on an ultrasound imaging examination can help medical experts exchange information and understand the conditions of patients from clinical perspectives [4]. The descriptive text recorded in ultrasound reports provides an additional source of clinical data to facilitate decision making during ultrasound examinations. However, the complexity of clinical data poses significant challenges to the practical application of computer-assisted analysis and modeling [5]. Medical data are annotated by experts in the field of medicine, with predictions made manually. In the absence of domain experts or a shortage of professional healthcare personnel, the erroneous interpretation of medical data can pose serious consequences for patients [6]. Thus, it is crucial to have a comprehensive understanding of novel knowledge extraction methods that are suitable for analyzing domain-specific texts to utilize them effectively [7,8].

Knowledge graphs use graph-based data models to capture knowledge in application scenarios that involve integrating, managing, and extracting value from various data sources [9]. The conversion of the narrative text in ultrasound reports into a knowledge graph form facilitates the rapid modeling and reuse of existing clinical knowledge in these historical reports. Accurate health information management plays a vital role in collecting relevant information on diseases, which, in turn, supports effective clinical decision making [10,11]. Knowledge graphs are valuable data structures in the field of information retrieval and representation, allowing for knowledge inference and prediction based on entity relationships. Advances in digital technology have opened up new possibilities for language processing, including natural language processing (NLP) methods [12,13] and tools capable of constructing free text and performing semantic analysis. Developing intelligent medical applications such as disease diagnosis and answering healthcare questions requires the critical task of representation learning for these graphs [14]. Therefore, leveraging the proper modeling techniques for knowledge graphs with ultrasound text can greatly facilitate medical decision making based on ultrasound reports.

Research on existing knowledge graphs has extensively investigated analytic techniques for converting natural text into entities, relationships, and semantic structures [15]. While many existing tools are proficient at analyzing general text [16], only a few are capable of extracting domain-specific knowledge in the medical field [17]. In addition, most of the existing knowledge graphs in the medical field are based on data from various open databases, the quality of which cannot meet the needs of disease diagnosis or treatment [18,19]. Most models are not easily transferable and reusable in non-English medical NLP models. Instead, retraining with new data in the target language is typically necessary [20]. Accurately and meaningfully extracting information from raw ultrasound reports poses a significant challenge due to the complex and unstructured nature of the text.

Furthermore, existing approaches involving the extraction of semantic relationships and entity information in electronic medical records mainly rely on rules with manual features or machine-learning models [21,22,23]. However, these methods have not fully exploited the hierarchical semantics of textual language morphology, including word dependencies and syntactic relationships [24], which can make it difficult to identify relevant clinical entities and terminology accurately. In addition, unstandardized physician writing habits can lead to limitations in knowledge graph scalability and accuracy. Methods with insufficient accuracy are difficult to model for decision making, especially when analyzing clinical texts [25]. Medical entities frequently use shorthand, abbreviations, and aliases in real-world scenarios [26]. The omission of subjects in the text presents a significant challenge when analyzing similar reports [27]. 

The objective of this study is to provide more accurate clinical knowledge for downstream knowledge inference tasks and facilitate computer-assisted clinical decision-making processes for ultrasound reports. The contributions of this study are as follows.

- Firstly, we propose a novel key term network extraction method for ultrasound reports that achieves the highly accurate decomposition and annotation of the narrative text within reports.
- Secondly, we introduce a knowledge representation framework based on ultrasound reports that provides a structured and intuitive visualization of ultrasound report knowledge.
- Finally, we propose a knowledge graph completion model to address the lack of entities in physicians’ writing habits and improve the accuracy of using ultrasound knowledge.

The Section 2 presents techniques and algorithms for extracting networks of key terms for ultrasound reports from natural language in the text. In addition, a framework for knowledge representation based on ultrasound reports is presented. The Section 3 presents experiments to evaluate the performance of the proposed method. The Section 4 discusses the application value and drawbacks of the proposed method. Finally, we summarize our research in the Section 5.

## 2. Materials and Methods

**Figure 1:**
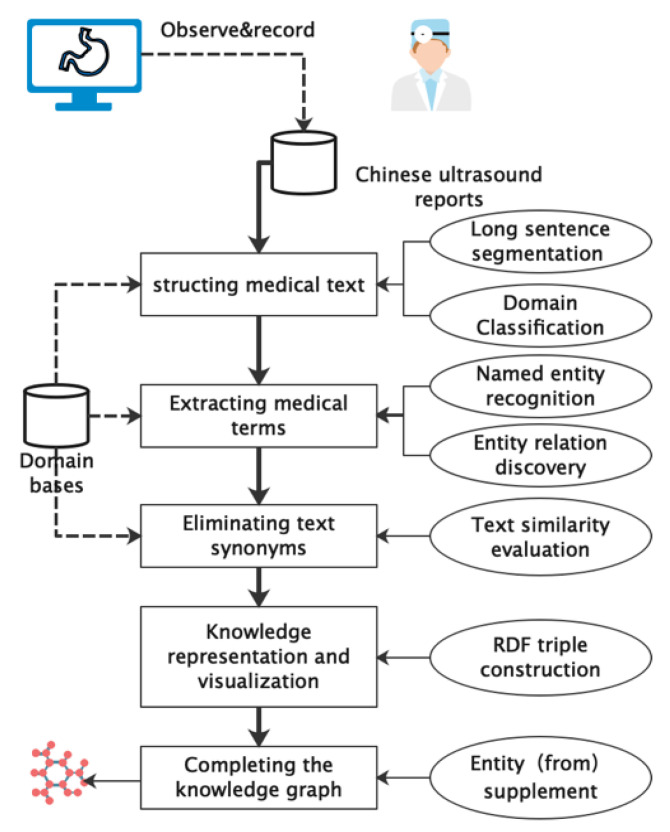
Overview of the analysis process in this study.

In this article, we present a comprehensive method for extracting and visualizing clinical knowledge from ultrasound reports. Our approach involves several stages of knowledge extraction to simplify the reports and improve their accuracy. In the first stage, we use a word segmentation strategy to structure the medical text and extract a term network (TN) from it. In the second stage, we apply specific syntax analysis rules to identify meaningful entities and the relationships between entities in the structured TN, generating a simple knowledge graph (SKG). In the third stage, we use the Word2Vec algorithm to extract multiple instances of the same entity and reduce confusion about grammar relations in the report to improve the accuracy of the structured results. In the fourth stage, we perform knowledge representation and visualization based on the simple knowledge graph extracted in the second stage. The constructed knowledge graphs can support knowledge discovery from ultrasound reports and facilitate computer-assisted decision making based on reports. Finally, we propose a fine-grained model called the knowledge graph completion model (KGCM) to supplement the knowledge graph by combining the importance of entities that are omitted. Overall, our proposed method offers a comprehensive approach to leverage knowledge from ultrasound reports and utilize knowledge graphs to facilitate computer-assisted decision making based on reports. The overview of the proposed approach is shown in Figure 1.

### 2.1. Structuring Medical Text

Ultrasound reports often contain complex descriptions for each organ that is an examination item, leading to numerous short sentences and relatively long overall sentence lengths. As the sentence length increases, the complexity of syntactic analysis also increases, resulting in more complicated grammar tree structures and a decline in the quality of the final syntactic analysis results. To address these challenges, we propose a method for dividing long sentences into multiple parts and processing them separately. By breaking down the sentences, we can extract the most effective information and improve the accuracy of our syntactic analysis. This approach allows us to handle the complexity of the ultrasound report’s language and improve the quality of our results. Furthermore, the knowledge sharing of exact terminology between experts in any sector requires domain-specific vocabulary [28]. This is particularly true in the medical field, where exact terminology is not present in general language dictionaries. To address this challenge, we aim to develop an algorithm that can represent basic concepts and their semantic relationships extracted from the text using TNs. Our algorithm preserves the order of sentences and words from the reports, allowing us to create network-based graphs that represent the report’s content. By utilizing these graphs, we can perform further analysis and gain a deeper understanding of the text. This approach helps to standardize the language used in the medical field and facilitate knowledge sharing between experts.

Our proposed algorithm consists of several key components designed to accurately extract and represent essential concepts and their semantic relationships. First, we use a word segmentation method to segment text from the report, which is denoted by the function WSM (*text*). Second, we use a function called SNLP (*w*) to return the result of *w* with its part-of-speech (*pos*) and normalized word (*n_w_*). SNLP, as a function, encapsulates various techniques such as syntactic analysis, *pos* labeling, and so on in order to extract the required information from *w*. The complete algorithm is illustrated as Algorithm 1. By using these functions together, we can accurately extract and represent the essential concepts and their semantic relationships.
**Algorithm 1**. Structuring narrative text from ultrasound reports**Input**: Narrative text of a report (*text*) **Output**: Term network of the report (*TN*) 1:    Let *t* ← a list of terms through the WSM (*text*) 2:    Let *s* ← an empty set of concept relationships between two concepts {*r*: (*c*_1_, *c*_2_)}, where a concept is defined as a tuple *c* = (*w*, *n*_w_, *pos*). 3:    for each *w* ∈ *t* perform the following: 4:       Let *ST* be the result from the SNLP (*w*) 5:          Let *pos* ← *ST.pos*
6:          Let *n_w_* ← normalize (*w*) from *ST* 7:          Let *c*(*t*) ← a tuple: (*w*, *n_w_*, *pos*) 8:    end for 9:    for ∀*c*_1_, *c*_2_ ∈ *t* perform the following: 10:     If dist(*c*_1_(*t*), *c*_2_(*t*)) = 1 then Append (*c*_1_, *c*_2_) into s 11:  end for 12:  return *TN* (*s*)

### 2.2. Extracting Medical Terms

Using the structured results from the *TN*, each ultrasound report is transformed into a set of report-based semantic models that support the following information extraction techniques. In this stage, a grammatical tree among words is obtained from the syntax analysis of the text in a report using the *TN*. We can obtain attributes and their corresponding values for entities in the text by analyzing the syntactic relationships and the corresponding part of speech in the tree. Finally, the resulting data from the ultrasonic-related text, described in natural language, is converted into an entity–attribute–value format and stored in a simple knowledge graph (SKG). The SKG is defined as a set of entity relations, denoted as {(entity, relation, entity)}. To achieve this, we defined rules to obtain named entities and implemented knowledge extraction methods to extract entity relations from complex texts. Table 1 presents the rules for entity relation extraction, along with the corresponding relationships for each rule. The complete algorithm is shown in Algorithm 2. By utilizing named entity recognition, predefined rules for information extraction, and transformation networks, we could extract relevant information from the text in a flexible and efficient manner.
**Algorithm 2**. Constructing simple knowledge graphs based on the term network**Input**: a term network of a report (TN = {*t*_1_, *t*_2_, *t*_3_, …}) **Output**: a simple knowledge graph (SKG = {*r*_1_, *r*_2_, *r*_3_, …}) 1:    Let *R* ← a set of {rule: (*tag*_1_, *tag*_2_, …, *tag*_c_} where *c* is the number of tags in a rule and tag ∈ {*pos*} ∪ {custom tag} 2:    Let *M* ← a subset of *TN* where ∀term ∈ *M* are restricted to rules *R*. 3:    for ∀*t*_1_, *t*_2_, *t*_3_, … *t*_n_ ∈ *M* perform the following: 4:       if rule of (*t*_1_, *t*_2_, *t*_3_, … *t*_n_) ∈ *R* in Table 1 begins 5:          Let *r* ← a tuple that preserves an entity relation in Table 1 6:          if *n* = 2 then *r* ← (*t*_1_, a predefined relationship, *t*_2_) 7:          if *n* = 3 then *r* ← (*t*_1_, *t*_2_, *t*_3_) 8:          if *r* is not null then Append *r* into *SKG* 9:       end if 10:  end for 11:  return *SKG*

### 2.3. Extracting Medical Terms

We then use the SKGs constructed in the previous steps to implement text synonym elimination based on textual similarities. To accomplish this, we utilize the Word2Vec algorithm [29] that is trained on high-frequency words in the ultrasound texts following word segmentation. We use cosine similarity to compare the semantic relevance among words, with a higher value indicating greater similarity. By comparing the cosine similarity between words, we can construct a synonym table that identifies and eliminates textual synonyms that could cause ambiguity in knowledge representation. Word2Vec is known for its ability to capture the meaning of words without relying on their syntactic structure and can identify semantically related words even if they are not grammatically similar. The Word2Vec algorithm is trained using a sliding window approach, which involves moving a window of fixed size over the text corpus and using the words within the window to predict the target word. After neural network training, we obtain a word vector space that contains both the location and semantic information. The distance between two vectors can be regarded as the semantic similarity between words and can be calculated using different formulas such as the Euclidean distance, Chebyshev distance, and Cosine similarity. Cosine similarity mainly emphasizes the variation in the direction of word vectors in the vector space, and this difference makes it more accurate in assessing the semantic relatedness between words compared to other methods. The closer the cosine value between two vectors is to 1, the closer the angle between them is to 0 in vector space, indicating that the words represented by these vectors are semantically similar. Our approach is highly efficient and accurate, leveraging the power of Word2Vec to represent the meaning of words and identify textual synonyms in the context of ultrasound-related knowledge representation.

### 2.4. Knowledge Representation and Visualization

Knowledge graphs provide a powerful way to represent complex clinical knowledge, and ontologies are an essential tool for constructing them. This is particularly valuable in the field of ultrasound imaging, where terminology and definitions can vary widely between different regions and specialties. By standardizing the vocabulary used to describe ultrasound findings, ontologies help ensure that clinical knowledge can be shared and understood across different contexts. The Resource Description Framework (RDF), as an ontology language, is a widely used tool to construct knowledge graphs using triples. Each type of knowledge in RDF is represented as (Entity (from), Relation, Entity (to)). In the context of ultrasound reports, RDF triples can be used to represent various aspects of patient health, such as the size and location of tumors, blood flow patterns, and abnormalities in organs or tissues. By organizing these entities and relationships into a knowledge graph, clinicians and researchers can better understand the complex interactions between different aspects of patient health and make more accurate diagnoses. Furthermore, ontologies and knowledge graphs provide a foundation for machine learning algorithms to extract and apply clinical knowledge, leading to improved decision making and patient outcomes. A triple of RDF is defined as follows:

a triple = (*e_f_*, *r*, *e_t_*), (1)

where *e_f_* and *e_t_* represent the vectors of the head and tail nodes, respectively. Based on Equation (1), an example of representing the knowledge of liver tissues using an ontology model is shown as follows:
<?xml version=“1.0”?> <rdf:RDF xmlns=“#”>        <owl:Ontology rdf:about=“”/>        <owl:ObjectProperty rdf:about=“#hasProperty”>               <rdf:type rdf:resource=“&owl;TransitiveProperty”/>        </owl:ObjectProperty>        <owl:ObjectProperty rdf:about=“#internal_diameter”/>        <owl:Class rdf:about=“#liver”/>        <owl:Class rdf:about=“#portal_vein”>               <rdfs:subClassOf rdf:resource=“#liver”/>               <owl:disjointWith rdf:resource=“#surface”/>        </owl:Class>        <owl:Class rdf:about=“#surface”>               <rdfs:subClassOf rdf:resource=“#liver”/>        </owl:Class>        <owl:Class rdf:about=“#trunk”>               <rdfs:subClassOf rdf:resource=“#portal_vein”/>               <rdfs:subClassOf>                      <owl:Restriction>                             <owl:onProperty rdf:resource=“#hasProperty”/>                             <owl:onClass rdf:resource=“#trunk”/>                             <owl:minQualifiedCardinality rdf:datatype=“&xsd;nonNegativeInteger”>1</owl:minQualifiedCardinality>                      </owl:Restriction>               </rdfs:subClassOf>        </owl:Class>        <owl:NamedIndividual rdf:about=“#no_smooth”>               <rdf:type rdf:resource=“#surface”/>        </owl:NamedIndividual> </rdf:RDF>

### 2.5. Completing Knowledge Graph

The expression of ultrasound reports can sometimes be incomplete, with hidden information omitted and the grammatical structure appearing relatively concise. Generally, experts often describe the same entity when it first appears and then omit it in the next sentence to simplify the description. This can result in confusion in entity-pointing relationships and missing entities in the constructed knowledge graph. To address this issue, we propose a knowledge graph completion model (KGCM) based on the importance of entities, which embeds triplet information into low-dimensional vector space using the TransH model to achieve knowledge computability. The importance of entities is then combined to supplement the knowledge graph of omitted entities and avoid confusion in the knowledge representation and visualization process.

In the TransH model [30], the relation *r* is represented by a hyperplane *w_r_* and a relation vector *d*_r_, with the relation *r* in a different embedding space with *e_f_* and *e_t_*. In each triple (*e_f_*, *r*, *e_t_*), *e_f_* and *e_t_* are projected on the hyperplane, corresponding to ${e_{f}}_{⊥}$, ${e_{t}}_{⊥}$, which can be defined as follows:

(2) $${e_{f}}_{⊥}=e_{f}-w_{r}{e_{f}}_{⊥}w_{r},$$

(3) $${e_{t}}_{⊥}=e_{t}-w_{r}{e_{t}}_{⊥}\;w_{r},$$

where ${e_{f}}_{⊥}$ and ${e_{t}}_{⊥}$ denote the mapping vectors that map *e_f_* and *e_t_* onto the hyperplane *w_r_*. $w_{r}{e_{f}}_{⊥}w_{r}$ denotes that *e_f_* is the projection on hyperplane *w_r_*. Additionally, it is important to note that the magnitude of the hyperplane vector *w_r_* is normalized to unity, as indicated by the following constraint:

(4) $${∥w}_{r}∥=1.$$

For a correct triple (*e_f_*, *r*, *e_t_*), we consider that in the vector space ${e_{f}}_{⊥}+$ *d*_r_ $\approx {e_{t}}_{⊥}$. So the score function *f*_r_(*e_f_*, *e*_t_) can be defined as follows:

(5) $$f_{r}\left(e_{f},\;{\;e}_{t}\right)=∥\left(e_{f}-w_{r}{e_{f}}_{⊥}\;w_{r}\right)+d_{r}-(e_{t}-w_{r}{e_{t}}_{⊥}\;w_{r}){∥}_{2}^{2}.$$

Equation (5) measures the squared distance between the sum of the projected embeddings and the corresponding relation vector. By minimizing this score function, the TransH model aims to capture meaningful relationships between entities in different embedding spaces.

TransH effectively embeds knowledge into low-dimensional vector space, enabling the computability of knowledge in knowledge graphs and supplementing *e_f_* in the knowledge graph triplet due to the lack of ultrasound report writing habits; however, it does not consider the importance of different entities *e_f_* to the same entity *e_t_*. To address this challenge, our KGCM incorporates a proximity function *k*(*e*_i_, *e*_j_), which evaluates the importance of an entity based on the distance between entities. The proximity function is defined using a Gaussian kernel, which converts the dot product in the infinite-dimensional space into a Gaussian function of the distance between points in the data space. This approach is particularly suitable for evaluating the importance of an entity based on the distance between entities. When the distance between *e*_i_ and *e*_j_ is small, the value of *k* decreases slowly. However, when the distance value is in the middle range, it drops rapidly, and then when the distance value between two nodes is very large, it drops slowly again. More formally, the proximity function *k*(*e*_i_, *e*_j_) can be defined as follows:

(6) $$k\left(e_{i},e_{j}\right)=e^{\frac{∥e_{i}-e_{j}{∥}^{2}}{{-2\sigma }^{2}}},$$

where *σ* is the bandwidth parameter, which plays a crucial role in determining the radial range of action of the Gaussian kernel function. Specifically, *σ* controls the local range of the Gaussian kernel function, thereby influencing the accuracy of the proximity function *k*(*e*_i_, *e*_j_) when evaluating the importance of an entity through the distance between entities. A smaller *σ* value restricts the range of the Gaussian kernel function to a smaller region, resulting in a higher weight for entities that are closer together. Therefore, selecting an appropriate value for *σ* is critical to ensure the accuracy and effectiveness of the KGCM model to complete knowledge graphs in ultrasound reports.

Based on Equations (5) and (6), the scoring function of the KGCM model is defined as follows:

(7) $$f_{r}\left(e_{f},\;\;e_{t}\right)=∥{e_{f}}_{⊥}+d_{r}-{e_{t}}_{⊥}{∥}_{2}^{2}\;\times \;e^{\frac{∥e_{f}-e_{t}{∥}^{2}}{{-2\sigma }^{2}}}.$$

Equation (7) combines the considerations of the TransH model with the proximity function to capture the relationships and importance between entities in knowledge graphs.

The KGCM model provides a novel approach to complete knowledge graphs in ultrasound imaging by supplementing omitted entities and considering the importance of different entities. This approach has the potential to significantly enhance the accuracy of diagnoses and facilitate more effective communication between clinicians and researchers.

## 3. Results

For this study, we obtained a dataset of ultrasound reports from a cooperative hospital in China. The dataset included abdominal examination results from 4484 patients with cirrhosis and ascites and contained 142,834 entity relationships with 12 different relationship types. An abdominal ultrasound examination is a common hospital examination that mainly explores the ultrasound characteristics of the abdominal tissue, including five main areas of liver, gallbladder, bile duct, pancreas, and kidney. Table 2 provides a record of the ultrasound reports used in our experiments.

### 3.1. Visualization of Ultrasound Reports

**Figure 2:**
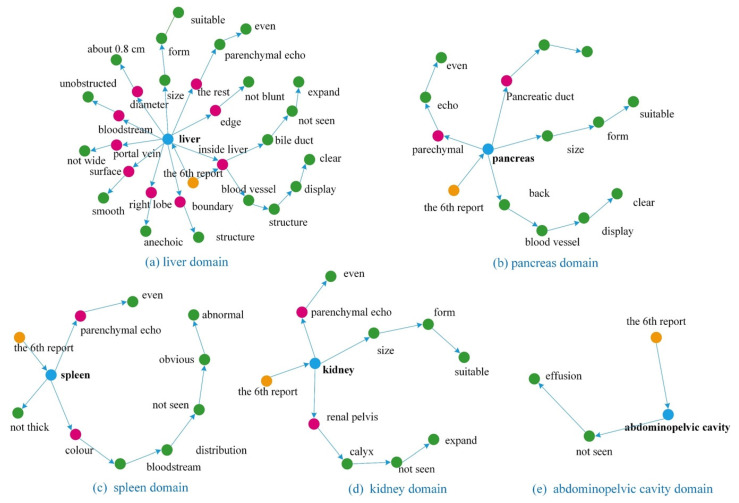
Group of semantic models to represent multiple domain-based narrative texts clustered in a report using our method.

**Figure 3:**
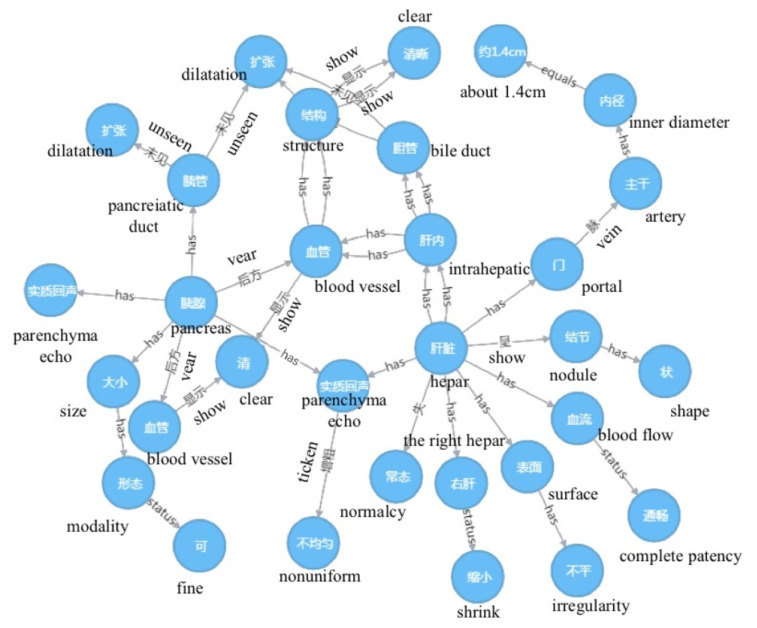
An example of knowledge graph representation for a report.

To evaluate the performance of the proposed method to facilitate the representation of knowledge for ultrasound reports, we conducted the experiments at different stages of the process. Specifically, we evaluated our approach to medical text structuring and knowledge representation techniques. Table 3 shows the results of the medical text structuring process, which involves segmenting long sentences based on syntactic and semantic features. The results are reported for TN, SKG, and SKG with the KGCM model. In addition, Figure 2 displays semantic models for analyzing the results of various organs from a report. The five domains presented in the figure depict the semantic relations between the components. We used knowledge graph representation techniques to visualize the ultrasound reports, which resulted in high-quality graphs. Figure 3 illustrates a domain-based knowledge graph defined in the RDF format, where missing entities were completed using the KGCM to enhance the accuracy and completeness of the graph.

### 3.2. Evaluation in Knowledge Extraction

**Figure 4:**
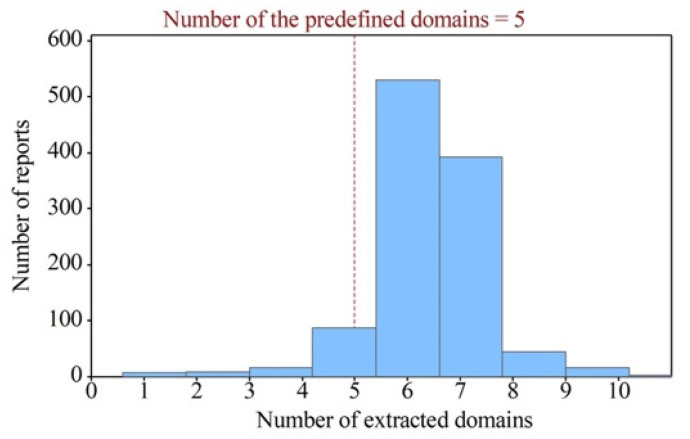
Distribution of the number of reports based on the number of extracted domains.

Based on a thorough investigation of the distribution of the number of extracted domains and entity relations from the ultrasound report dataset, we provide a comprehensive evaluation of the proposed ultrasound report text information extraction method. Figure 4 shows a distribution of the number of reports based on the number of extracted domains. The results using our method show that the number of extracted domains from the reports exceeded five, indicating that our proposed method automatically extracts appropriate domains based on the semantics of the reports rather than relying on a fixed and predetermined set of domains for analysis. This approach leads to a more accurate and comprehensive knowledge representation of ultrasound reports.

**Figure 5:**
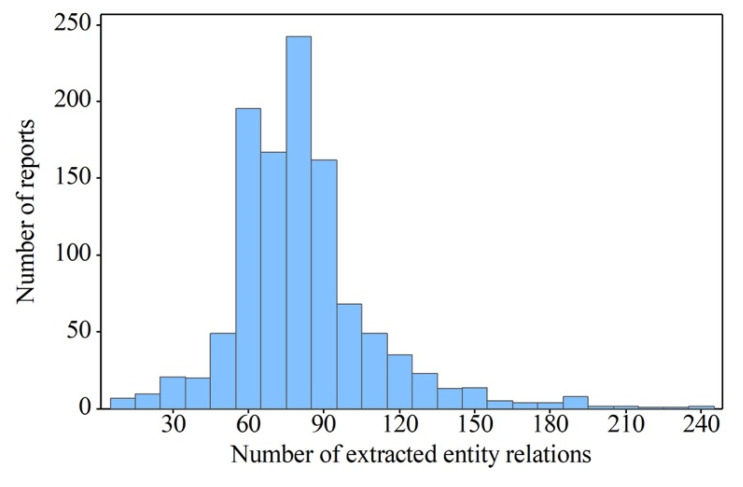
Number of extracted entity relations using our method.

Table 4 shows the results of the commonly used relationship formulas extracted from all the datasets. In addition, to evaluate the performance of our entity–relation extraction method, we analyzed the distribution of the number of extracted entity relationships in the report dataset. The results are shown in Figure 5, demonstrating a high frequency of extracted entity relations that were relevant and significant in ultrasound reports. These relations can serve as templates to standardize physicians’ writing habits and improve the quality of medical decision making.

To assess the effectiveness of our proposed structured method to construct ultrasound reports, we conducted experiments to compare it with two commonly used methods in the scientific literature: the typical text-matching method and the pattern-matching method. The text-matching method relies on a fixed set of five organ-related domains to extract information from the report, which results in a static outcome. The pattern-matching method employs simple rules of regular expression to extract information, but it may miss important details that do not fit the predefined patterns. To evaluate the performance of our method, we used a set of widely recognized information retrieval metrics to assess our model’s performance, including precision (P), recall (R), and the F1 score (F1). We obtained accurate values for P, R, and F1 through manual annotations by medical experts. Furthermore, we defined an evaluation index (*η*) that considered the number of extracted domains (*n*), the number of valid domains (*v*), and the baseline (text-matching method). This metric allowed for the effective extraction of domains while measuring the performance of information changes. A larger value of *η* indicates more meaningful overall extracted domains and better performance in capturing relevant information from ultrasound reports. The formula for *η* is given as follows:

(8) $$\eta =\frac{v}{baseline*(v/n)}.$$

**Figure 6:**
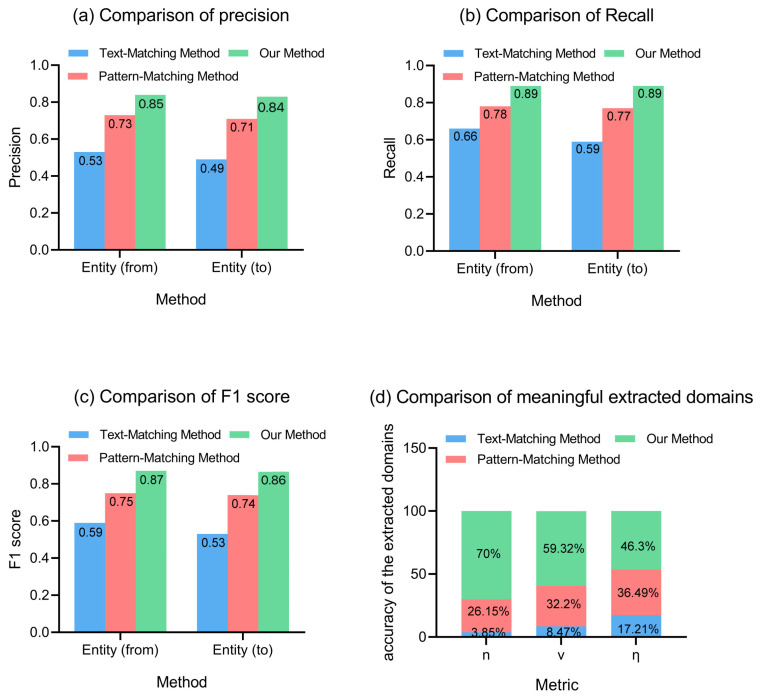
Comparison of model performance with different methods.

As shown in Figure 6, our proposed method outperforms the text-matching method and pattern-matching method on the ultrasound report dataset, achieving the highest P, R, F1, and *η* scores. This indicates that our method can identify most medical entities in the unstructured text, thereby achieving the desired goal of automated extraction. The Entity (from) category was found to be recognized more accurately than the Entity (to) category due to the variation in sentence structures arising from different doctors’ writing styles. While incomplete sentence structures posed a difficulty for the baseline method, our method overcame this through semantic rules and entity supplementation, identifying them as *TN*. These results show that our proposed method for the text structuring and information extraction of ultrasound reports can effectively extract relevant medical terms and their terminological relationships, leading to a reduction in the semantic complexity and improved analysis of ultrasound reports.

### 3.3. Evaluation in Knowledge Expression and Visualization

In this stage, we aim to implement the elimination of textual synonyms using the Word2Vec tool. To identify synonyms, we performed cosine similarity computation between pairs of words, such as “unsmooth” and “Rough”, as presented in Table 5. It was observed that “unsmooth” occurred quite frequently in this set of synonyms; thus, it was deemed fitting to be utilized as the category name. Consequently, we replaced all other synonyms belonging to this category that were present in the text of the abdominal routine ultrasound report with the term “unsmooth”. By using synonym sets, we were able to unify synonymous expressions in ultrasound reports, thereby eliminating the problem of multiple expressions for a single concept. 

Furthermore, to evaluate the effectiveness of our proposed KGCM model and compare it with other state-of-the-art methods, including TransH [30], TransE [31], TransR [32], RotatE [33], and EEM-CMR [34], we empirically illustrate the importance of knowledge representation and inferring relational patterns for the task of predicting missing entities. TransE [31] embeds entities and relations uniformly into low-dimensional feature space, treating relations as translation vectors between head and tail entities. TransR [32] extends relation-specific hyperplanes to relation-specific spaces, defining projection matrices from entity vectors to relation spaces. RotatE [33] defines each relation as a rotation from a starting entity to a target entity in complex spaces. EEM-CMR [34] extracts partially missing entities heuristically by setting up segmentation reorganization rules.

In addition, to evaluate the method proposed in this paper, we used the standard dataset for NCBI disease [35], JLNPBA [36], and our dataset of Chinese ultrasound reports. NCBI disease [35] contains 793 PubMed abstracts, 6892 disease mentions, and 790 unique disease concepts. JLNPBA [36] was formed from MEDLINE using the MeSH terms “human,” “blood cell,” and “transcription factor” and contains 2000 abstracts that are manually annotated. To evaluate the performance of complementing missing entities, we used metrics such as average precision, recall, and F1 scores across all entity types.

Table 6 shows that KGCM outperforms other methods, with higher P, R, and F1 on all three datasets. This indicates that KGCM is more effective at handling triadic relationships, enabling knowledge acquisition, fusion, and inference. All methods perform well in the ultrasound report dataset since the ultrasound report dataset eliminates a large number of inverse relationships as well as inverse relationship triples, which helps the dataset to distinguish the performance of the models more efficiently. Our study underscores the crucial role of preprocessed data in improving the accuracy of algorithm used in medical data analysis for KGCM and similar models. 

## 4. Discussion

We present a robust and comprehensive method for structuring text, information extraction, and knowledge representation in ultrasound reports. This method extracts entities, relationships, and values from the report and utilizes the entity importance-based KGCM model to supplement implicit information not explicitly stated in the sentences of the report. Our experimental results validate the effectiveness of this method in terms of knowledge extraction, representation, and visualization, offering a promising approach for leveraging the knowledge embedded in ultrasound reports to aid medical professionals in their decision making. The proposed method has significant potential for accelerating the development of computer-aided analysis tools, leading to more efficient and accurate medical decision making.

Our proposed method outperformed traditional methods when extracting knowledge from complex ultrasound reports with a significantly higher extraction index η of 2.69 compared to the general pattern matching method (2.12) and general text matching method (1.00), as well as achieving the highest P (0.85), R (0.89), and F1 (0.87) when evaluated on the ultrasound report. Additionally, our entity relation extraction method is highly effective from a clinical perspective. Our proposed method utilizes knowledge graph visualization to analyze ultrasound reports, and the results presented in Table 4 indicate that we extracted a significant amount of clinical knowledge from the ultrasound reports. For example, changes in size and form were found to be the most frequent entity relationships and were of particular concern to patients with hepatic ascites caused by liver cirrhosis. This demonstrates the meaningfulness of the high-frequency entity relationships we extracted in the context of the ultrasound reports. 

To further improve the quality of our results, we employed a knowledge graph model called KGCM based on entity importance to supplement the implicit information omitted in the ultrasound report. The experimental results in Table 6 indicate that the supplementation of missing entities through knowledge graph embeddings yields better performance compared to the token-based recombination approach, as exemplified by EEM-CMR. Notably, the success of KGCM in outperforming other knowledge graph embedding methods, particularly in the ultrasound report dataset, underscores its potential for applications where accurate and comprehensive entity completion is paramount. Moreover, the relative improvements achieved by KGCM in F1 scores, when compared to the latest knowledge graph-based method RotatE, signify its robustness across different datasets. The notable enhancements in F1, specifically 3.7%, 2.6%, and 4.8% in the NCBI, JLNPBA, and ultrasound datasets, respectively, demonstrate the generalizability and effectiveness of KGCM in diverse biomedical contexts.

One potential limitation of our approach is the fact that the knowledge extraction rules we defined are focused on Chinese ultrasound reports and are characterized by a narrative style different from that of English reports. Therefore, to accommodate the unique semantic features of English reports, it may be necessary to make some modifications to our detailed text structuring techniques. Nevertheless, the text structuring methods, semantic models, text synonym elimination, and KGCM proposed for analyzing ultrasound reports can be applied to any language. Another limitation is that our method can only analyze textual data. As ultrasound report data sources include both text and images, the accuracy of our method with respect to data from other sources cannot be guaranteed. Further multimodal analysis incorporating image data has the potential to optimize the model and enhance its robustness and generalization capabilities.

## 5. Conclusions

Our study proposes a knowledge graph-based approach for constructing, extracting, and representing knowledge within ultrasound reports. We propose a network extraction method for the key terms of ultrasound reports to extract entities and relationships from ultrasound reports and obtain reliable clinical knowledge. Moreover, we developed a knowledge graph for ultrasound reports, addressing the issue of missing entities caused by irregular writing habits using the KGCM method. In comparison to other baseline methods, our approach achieves the highest P (0.85), R (0.89), and F1 (0.87) across three testing datasets, providing evidence for the effectiveness and practicality of the knowledge graph.

Future research directions for this study involve the integration of additional multimodal data, such as images, to enhance the reliability and completeness of the knowledge graph. Furthermore, there is a need for the thorough validation of the extracted knowledge graph in real clinical environments to ensure its accuracy and applicability in practical medical settings. If the proposed method proves capable of generating reliable knowledge graphs, there is the potential to establish new clinical decision support applications in diagnosis and treatment.

## Figures and Tables

**Table 1:** Rules of entity relation extraction.

Id	Rules	Relationships
1	/organ→/verb→/adj	(/organ, /verb, /adj)
2	/organ→/adj	(/organ, show, /adj)
3	/organ→/verb	(/organ, show, /verb)
4	/organ→/n→/adj	(/organ, has, /n) (/n, show, /adj)

**Table 2:** Example of main items in ultrasound reports.

Item of Report	Definition	Example Content
patientid	Patient ID	TG923088WY
admiss_times	Examination frequency	1
StudyClass	Types of ultrasound examinations	Abdominal routine
Observations of imaging	The condition of the organ tissues is observed and recorded by the doctor through operating the ultrasound device	The liver is abnormal, and the right liver is reduced with an uneven surface, the substantial echo is thickened, uneven, and nodular, and the hepatic vascular structure is clear. The internal diameter of the main portal vein is 1.4 cm, and the blood flow is unobstructed. The intrahepatic bile duct does not expand. The size and shape of the pancreas are observed, the echo is homogeneous, the pancreatic duct is not dilated, and the posterior pancreatic vessels are clear. The splenic thickness and length are approximately 4.6 and 10.7 cm, the echo is homogeneous, and the color blood flow distribution is normal. The size and shape of the double kidneys are homogeneous, the echo is homogeneous, and the renal pelvis, calyx, and ureter do not expand.
Result of examination	The ultrasound examination conclusion provided by the doctor	Liver cirrhosis, and portal hypertension are observed; the accessory umbilical vein is open; the spleen is slightly large.

**Table 3:** The results of knowledge extraction from ultrasound reports at different stages.

Original Text	TN	SKG	SKG with KGCM
The liver is abnormal, and the right liver is reduced with an uneven surface, the substantial echo is thickened, uneven, and nodular, and the hepatic vascular structure is clear.	liver; has no normalcy	{liver, has no normalcy}	{liver, has no normalcy}
	right liver; shrink;	{right liver; shows shrink;}	{right liver; shows shrink; }
	surface; uneven	{@, surface, uneven}	{liver, surface, uneven}
	substantial echo; thickens; nonuniform;	{substantial echo, thickens, nonuniform}	{substantial echo, thickens, nonuniform}
	show; nodule shape	{@, show, nodule shape}	{substantial echo, show, nodule shape}
	Intrahepatic; blood vessel; structure; show; clear	{liver, has, blood vessel}; {blood vessel, has, structure}; {structure, show; clear}	{liver has, blood vessel); {blood vessel, has, structure); {structure, show; clear}

**Table 4:** Entity relations extracted from the reports using our method.

Entity 1	Relationship	Entity 2	%
size	has	form	3.14
form	status	suitable	2.99
liver	has	inside liver	2.88
inside liver	has	blood vessel	2.37
parenchymal	echo	average	2.31
colour	has	blood stream	1.96
inside liver	has	bile duct	1.96
bile duct	unseen	expand	1.88
gallbladder	has	intracavity	1.68
blood vessel	has	structure	1.38
renal pelvis	has	calyx	1.15
outside liver	has	bile duct	1.12
gallbladder	has	wall	1.11
liver	has	parenchymal	1.11
spleen	has	parenchymal	1.07
parenchymal	status	echo	1.02
gallbladder	has	outside liver	1.00
liver	has	envelope	1.00
size	has	form	3.14

**Table 5:** An example of synonym elimination.

Semantically Similar Words	Cosine Similarity
Unsmooth	0.937705
Rough	0.851965
Less smooth	0.816471
Uneven	0.74689
Generally flat	0.588166

**Table 6:** The comparison of different methods on the NCBI, JLNPBA and ultrasound reports dataset.

Methods	NCBI			JLNPBA			Ultrasound Reports		
	P	R	F1	P	R	F1	P	R	F1
TransE	0.75	0.77	0.76	0.73	0.72	0.73	0.80	0.82	0.81
TransH	0.76	0.74	0.75	0.77	0.79	0.78	0.84	0.86	0.85
TransR	0.78	0.79	0.79	0.74	0.72	0.73	0.83	0.84	0.84
RotatE	0.82	0.81	0.81	0.78	0.75	0.77	0.81	0.84	0.83
EEM-CMR	0.76	0.75	0.76	0.71	0.70	0.71	0.82	0.81	0.82
KGCM	0.83	0.84	0.84	0.78	0.80	0.79	0.85	0.89	0.87

## Data Availability

The data presented in this study are available on request from the corresponding author.
